# Centromere Stability: The Replication Connection

**DOI:** 10.3390/genes8010037

**Published:** 2017-01-18

**Authors:** Susan L. Forsburg, Kuo-Fang Shen

**Affiliations:** Program in Molecular & Computational Biology, University of Southern California, Los Angeles, CA 90089-2910, USA; kuofangs@usc.edu

**Keywords:** replication, centromere, heterochromatin, fragile site, Swi6, Fork Protection Complex, cohesion

## Abstract

The fission yeast centromere, which is similar to metazoan centromeres, contains highly repetitive pericentromere sequences that are assembled into heterochromatin. This is required for the recruitment of cohesin and proper chromosome segregation. Surprisingly, the pericentromere replicates early in the S phase. Loss of heterochromatin causes this domain to become very sensitive to replication fork defects, leading to gross chromosome rearrangements. This review examines the interplay between components of DNA replication, heterochromatin assembly, and cohesin dynamics that ensures maintenance of genome stability and proper chromosome segregation.

## 1. Introduction

The centromere is the structural domain on the chromosome required for proper attachment of the spindle (reviewed in [1]). Disruption in centromere function is associated with numerical chromosome instability (nCIN). It is increasingly clear that the centromere is a fragile site prone to structural instability (sCIN), particularly in cancer cells [2,3,4]. Defects in chromosome segregation can contribute to centromere-linked breaks and fusions (e.g., [5]).

A source of stress may be the repetitive DNA sequences in the pericentromere (reviewed in [6,7]). Repetitive sequences throughout the genome are often fragile sites during replication (e.g, [8,9,10,11]; reviewed in [12]). Silenced heterochromatin may provide partial protection against repeat rearrangement [6,13,14]. Indeed, heterochromatin repeats are destabilized in cancer cells [2,3], and loss of the heterochromatin protein HP1 (*Sp*Swi6) is associated with cancer (reviewed in [15,16]).

In fission yeast, the heterochromatin structure is transiently disrupted during mitosis and re-established during the S phase [17,18]. The centromere replicates early in the S phase, and this timing depends on Swi6 protein [19,20,21]. Swi6 is also essential for the recruitment of cohesin to the centromere, which is required for proper kinetochore attachment and chromosome segregation [22,23]. Interestingly, both early replication and cohesion depend on the replication kinase DDK (DBF4 dependent kinase) [19,24]; thus replication dynamics are intimately involved in centromere function. Destabilizing the replication fork in cells lacking Swi6 enhances rearrangements and chromosome loss [14].

Together, these observations emphasize that the centromere is a fragile element in the genome. Thus, there must be pathways to manage intrinsic stress and prevent centromere-driven instability. This review describes work largely from the fission yeast *Schizosaccharomyces pombe*, to examine how replication progression and centromere structure interact to maintain genome stability in this region.

## 2. DNA Replication

### 2.1. Assembly and Activation of the Replisome

The initiation of replication in eukaryotes is highly conserved and depends on the sequential assembly of proteins that specify potential origins (see other reviews in this issue; also [25,26]). The origin is initially marked by ORC, the origin recognition complex. ORC serves as a platform for the Cdc18 (Cdc6) and Cdt1 proteins, which in turn load the heterohexameric MCM helicase complex. Together these form the pre-Replication Complex, or preRC, which is assembled and poised for activation in late M or G1 phase.

The activation at individual origins depends on the contributions of two kinases, the cyclin-dependent kinase CDK and DDK. The cyclin dependent kinase CDK conveys a global cell cycle signal to initiate the S phase, while the Dfp1/DBF4-dependent DDK kinase activates the individual preRCs by phosphorylating MCM proteins and other substrates (reviewed in [27,28]). This activation allows recruitment of additional proteins Cdc45 and the GINS complex, which together convert the preRC into an active helicase called CMG (Cdc45-MCM-GINS) that travels with other components to form the replisome [29]. CMG makes direct contact with DNA polymerase ε, which is the processive leading strand polymerase [30,31].

Stability of the replisome requires that the unwinding activity of the helicase is coupled to the leading and lagging strand polymerases to prevent excess unwinding ahead of DNA synthesis (reviewed in [32]). Mrc1 is a nonessential component of the replisome that couples the leading strand polymerase [33,34]. It is part of a complex that includes Swi1 and Swi3 (Hs Timeless-Tipin, Sc Tof1-Csm3, also called the Fork Protection Complex or FPC; reviewed in [35]). Similarly, the DNA polymerase α/primase complex that initiates lagging strand synthesis is coupled to CMG via a trimeric protein called Mcl1 (Sc Ctf4, Hs AND-1) [36,37]. Together, these components ensure that DNA synthesis and unwinding are coordinated.

### 2.2. DNA Replication Stress Causes Genome Instability

Disruptions in the smooth progression of DNA synthesis can be caused by intrinsic stresses such as late replicating regions, repetitive sequences, or replication/transcription collisions (reviewed in [38]). The genome regions that undergo stress may vary in different cell types, or be epigenetically modified; they often define chromosome fragile sites (CFS) that are particularly prone to breakage [39]. Breaks at CFS regions may be enhanced by low density of origins or defects in replication progression [12]. External insults also induce stress; these include drugs that inhibit DNA replication, disruptions in the ribonucleotide metabolism, or oncogene activation [38,40,41].

A common feature of replication stress is the presence of increased single-strand DNA (ssDNA; [38,42]). This can result from uncoupling the helicase from the polymerases (e.g., [43,44]), which leads to the accumulation of excess ssDNA, allowing the potential for fork regression [45] as well as resection (e.g., [46]). There is evidence that ssDNA can evade checkpoints, leading to abnormal mitosis, lagging chromosomes, and anaphase bridges [47,48]. Accumulation of ssDNA is also associated with increased rates of clustered point mutations [49]. The cell uses the ssDNA-binding protein RPA (Replication Protein A) to monitor levels of ssDNA, and its presence contributes to the cell’s damage response [42,50]. If RPA levels are reduced, DNA breakage occurs [51]. Thus, the amount of ssDNA produced during stress helps to modulate the appropriate response.

The classic cell cycle model suggests that that accumulation of ssDNA and replication stress activate a checkpoint signaling cascade that arrests the cell cycle and promotes repair and recovery [52,53]. There are multiple pathways to recover the fork [38,40,54,55]. For example, cells may reprime an existing fork or restart it by recombination following fork regression. They may undergo lesion bypass by template switching; or they may activate dormant origins to provide a ‘rescue replisome’ to ensure replication of the fragile region. Homologous recombination proteins such as Rad51 have a key role in the restoration of the fork, even in the absence of breaks [40,54]. If the fork cannot be restarted, it is said to collapse, generating double strand DNA breaks (DSBs), which can lead to chromosome rearrangements and mutations (e.g., [49,56]).

Persistent replication stress can result in DNA synthesis ongoing into mitosis and also generates abnormal chromosome segregation, which leads to loss of genome integrity [57]. Thus, a primary cause of death in replication-stressed yeast cells is not so much failure to replicate, as it is the attempt to divide with improperly replicated chromosomes (e.g., [58,59]).

## 3. Centromere Dynamics

### 3.1. Centromere Structure

**Figure 1:**
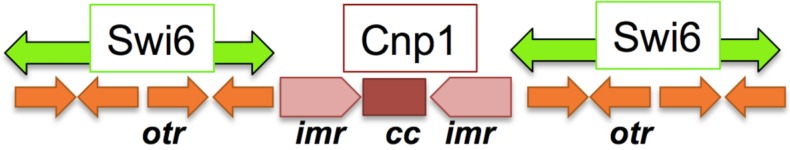
*S. pombe* centromere organization, in which heterochromatin protein Swi6 binds in the outer repeats flanking a central core with the centromere-specific histone Cnp1.

Most eukaryotic centromeres are large DNA elements that include highly repetitive sequences packaged into structurally rigid heterochromatin (reviewed in [1,60]). This surrounds a central region marked by the centromere-specific histone H3 variant CENP-A (*Sp*Cnp1). Fission yeast centromeres are large elements that adhere to the typical eukaryotic model. Each contains a unique central core sequence (*cc*) containing Cnp1^CENP−A^, flanked by two sets of repetitive sequences; the inner repeat (*imr*) unique to each centromere, and the outer repeats (*otr*), which contain multiple copies of the repetitive sequences *dg*, *dh*, and *cen253*, which are found in all three centromeres (Figure 1; [61]).

**Figure 2:**
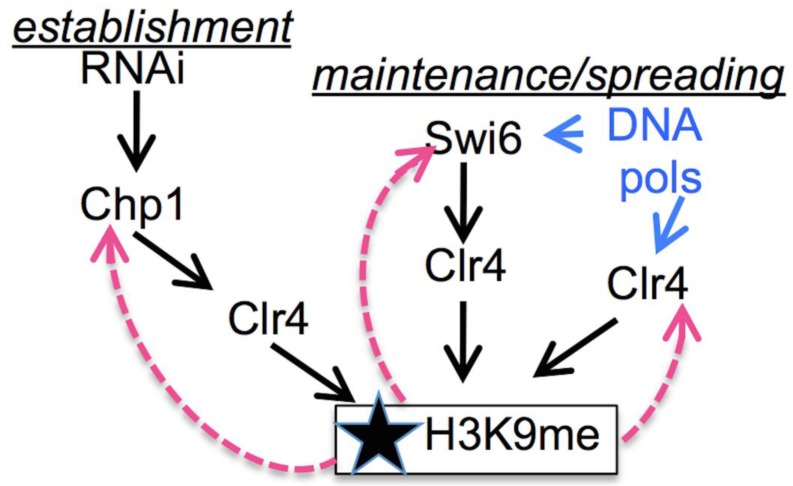
Multiple pathways contribute to the establishment and maintenance of H3K9 methylation. Pink dashed arrows indicate the binding of chromodomain-containing proteins to H3K9me. Both Swi6 and Clr4 bind DNA polymerases.

Heterochromatin at *otr* is defined by the presence of methylated histone H3K9. This histone methyl-mark is established and maintained by the methyltransferase Clr4^SuVar3−9^ [62,63] (Figure 2). Unexpectedly, Clr4 is targeted to this domain by transient de-silencing during G1 and the S phase. This allows a brief wave of convergent transcription to produce short non-coding RNAs [17,18]. These are processed by RNAi mechanisms and used to target Clr4 back to the site of transcription, re-establishing the methyl mark on newly incorporated histones [64,65]. This targeting requires the chromodomain (CD) protein Chp1, which binds H3K9me with high affinity and, as part of the RITS complex, associates with siRNA [65,66,67]. *chp1∆* causes a severe reduction in H3K9me but does not eliminate it entirely [68,69,70]. Finally, Chp1 is replaced by Swi6^HP1^, which also binds H3K9me through its conserved chromodomain to establish a transcriptionally silenced structure, required for efficient chromosome segregation (reviewed in [60]).

In addition to its association via Chp1, Clr4 also interacts with Swi6 to promote the spreading of H3K9me, and, via its own chromodomain, it can bind H3K9me directly [71,72,73]. Association between Clr4 and the leading strand DNA polymerase ε [74] provides a mechanism to couple histone modification directly to the replication fork. Together, this ensures that levels of H3K9me increase as the S phase proceeds [17,18]. This is a very simplified summary, as additional players that fine-tune the response continue to be identified (reviewed in [1,60,75]).

Cells with mutations in *swi6∆*, *chp1∆*, or *clr4∆* have moderate-to-severe silencing defects in the pericentromere, defects in chromosome segregation such as lagging chromosomes and chromosome loss (nCIN), and sensitivity to the spindle poison TBZ (e.g., [76,77,78,79]). Curiously, while *swi6∆* and *clr4*∆ affect other heterochromatin domains in the cell, *chp1∆* phenotypes appear centromere-specific, although the protein is associated with other regions [79,80].

### 3.2. Early Replication in the Centromere

**Figure 3:**
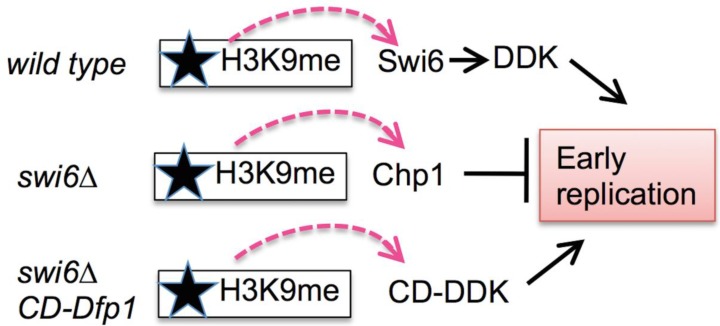
Model for replication timing.

The pericentromere contains numerous replication origins, which overlap with the transcription units that generate the siRNAs [81,82]. Unlike most heterochromatin, the fission yeast *otr* region undergoes replication early in the S phase [21]. This depends upon Swi6 [19,20], which is recruited to the centromere shortly after mitosis [80]. Swi6 binds the DNA replication initiation kinase DDK through the kinase regulatory subunit, Dfp1 [24]. Disruption of the interaction between Swi6 and Dfp1 leads to late replication, and artificially tethering Dfp1 to the chromatin via the Swi6 chromodomain restores early replication in *swi6∆* cells [19], suggesting that Swi6 recruits DDK to help activate early replication in the centromere domain (Figure 3). Importantly, this suggests that there is residual histone methylation remaining early in the S phase to be able to recruit chromodomain-containing proteins. Swi6 also associates with the origin binding proteins Cdc18^CDC6^ and ORC and with DNA polymerase α ([20,83]; and unpublished data); these observations place Swi6 at the preRC and at the fork.

Somewhat paradoxically, ChIP analysis suggests that most of the Swi6 is removed from the centromere during mitosis and largely returns in the late S phase [17,18]. Thus, there may be waves of Swi6 recruitment, with the second wave linked to the passage of the replication fork (e.g., via CAF1; [84]) and bulk DNA synthesis.

Interestingly, DDK recruitment by Swi6 is not essential for early replication in the absence of histone methylation, because the *clr4∆* mutant that blocks histone methylation replicates early [19,20]. Early replication is also observed in *chp1∆* mutants, but other mutations that significantly reduce H3K9me, such as the RNAi components *dcr1∆*, *hrr1∆*, or *rdp1∆*, cause late replication similar to *swi6∆* [80]. This may reflect residual H3K9me and Chp1 binding in RNAi mutants [85,86], leading to the suggestion that it is not H3K9me per se but the Chp1 bound to it that results in late replication in this domain [87]. In this model, recruitment of ectopic DDK either directly antagonizes Chp1 or overcomes an inhibitory effect of Chp1 binding on replication origin activation (Figure 3).

Components of the replisome have been directly linked to heterochromatin maintenance. The DNA polymerase ε subunit Cdc20 is associated with the Rik1 methylation complex, and, in its absence, silencing and histone methylation are reduced [74]. The lagging strand DNA polymerase α (Swi7) and its coupling factor Mcl1 are also required for normal silencing and interact with Swi6 [83,88]. Thus, proteins that write or read the histone methylation mark are directly linked to fork progression.

There may be a mechanistic requirement for early replication in the centromere domain. In *S. cerevisiae*, this is proposed to facilitate proper sister-kinetochore bi-orientation [58,89]. There is no Swi6 to recruit DDK in *S. cerevisiae*, and evidence suggests that the kinase is recruited to the vicinity by its association with the kinetochore [90]. Failure to replicate properly leads to breakage and abnormalities during chromosome segregation in budding yeast and other species [4,58]. Early replication in the centromere may also be linked to the recruitment of cohesin in this domain, which is essential for proper segregation (discussed below).

### 3.3. Genome Stress in the Centromere

The heterochromatic pericentromere has been associated with replication stress-induced breaks and rearrangements [91,92]. The pericentromere is made up of repeated sequences, and such sequences are known to be prone to recombination or replication fork pausing (e.g., [8,9,10]). From the M to the S phase, heterochromatin in the centromere is partly disrupted to allow transcription and siRNA production [17,18], creating a window of vulnerability. The unmasking of heterochromatin repeats during the S phase and leads to potential collisions between DNA and RNA polymerase. The RNAi proteins contribute to RNA polymerase eviction to reduce this possibility [82].

Even in normal fission yeast cells, there is evidence for constitutive low levels of damage at the centromere, which leads to the phosphorylation of histone H2A(X) in the *otr* repeats [93]. This modification is typically associated with double strand breaks [94,95]. The SMC5/6 protein complex, which is associated with genome maintenance during replication stress, is enriched at the centromere and other repetitive domains [96,97,98,99]. Brc1, a BRCT-motif containing protein that binds γH2A(X) and also contributes to replication fork restart, is likewise enriched at the centromere, where its presence depends on Clr4 [100]. There are tRNA genes flanking the centromere repeats that act as barriers to heterochromatin spreading [101,102,103]. tRNAs are also known to be replication fork barriers [104], so these natural pause sites may create intrinsic fragile domains even in an unperturbed S phase. Between natural replication fork barriers and repetitive sequences, the pericentromere seems primed for instability. There may be a requirement for this as, intriguingly, replication stress has been suggested to contribute to heterochromatin assembly (reviewed in [105]). In addition, despite the very different structure of centromeres in the budding yeast, there is evidence for constitutive fork pausing, a form of replication stress, in that system as well [106].

Recent studies suggest additional candidates that help to preserve the integrity of the pericentromere domain. Fission yeast has three homologues of the centromere associated protein Cenp-B; Abp1, Cbh1, and Cbh2 [107,108]. This is an ancient family thought to derive from a transposase that is shared in most eukaryotes but missing from budding yeast [109]. Cenp-B homologues have been linked to origin binding and to centromere maintenance [107,108,110,111,112]. Importantly, they are also involved in resisting rearrangements at long terminal repeats (LTRs) that are associated with transposons throughout the genome [113,114]. The Cenp-B proteins act antagonistically with a sequence-specific binding protein associated with fork arrest, Sap1 [113,115,116]. Sap1 is essential for viability, with mutants suffering centromere fragmentation and other evidence for genome instability [117,118]. Its functions are also linked to the FPC proteins Swi1 and Swi3 [119], which in turn are associated with replication of repetitive domains [120]. These complex interactions suggest that an additional function of the Cenp-B homologues at the centromere may be in countering the effects of stalled forks at the repetitive sequences of the outer repeats. It will be interesting to investigate more directly the role of fork stability in centromere integrity and heterochromatin assembly.

Importantly, fork stability mechanisms and heterochromatin work together to restrain instability. Heterochromatin is known to be refractory to recombination [121,122], and the kinetochore itself is also proposed to limit recombination in some systems [123]. Loss of heterochromatin proteins Swi6 or Chp1 causes synthetic growth defects and increased genome rearrangements especially when the replication fork is also destabilized, e.g., by a loss of the FPC [14]. Thus, replication fork processsivity associated with the FPC is of increased importance when repeated domains are destabilized.

However, there is evidence that recombination also contributes to the normal maintenance of the inner repeats *imr* that flank the central core. The inner repeat is conserved on either side of the core but is distinct in different centromeres [61,124] and may form a loop [125], leading to the suggestion that recombination mechanisms may preserve this domain [126]. Consistent with this, a study of a minichromosome derived from chromosome 3 identified the formation of isochromosomes, formed via recombination in the *imr* repeats [127,128,129]. The loss of recombination proteins Rad51 and Rad54 lead to an increased likelihood of rearrangement in this domain, which is dependent on the Mus81 endonuclease [127,128,129]. Mus81 is thought to convert fragile sites to double strand breaks [130,131], although it is unclear whether that is related to its function promoting centromere rearrangements. There are synthetic growth defects between *swi6∆* and *rad51∆* or *mus81∆* [14,82,132], which suggests that the mechanisms associated with recombination become particularly important when heterochromatin formation is impaired; again, this is consistent with a model in which heterochromatin opposes genome rearrangement.

## 4. Cohesion

Centromeres of sister chromatids are held together by two mechanisms (reviewed in [133,134]). The first is cohesin, a ring-shaped protein complex that is activated during replication to link newly synthesized sister chromatids together. The centromere is highly enriched in sister chromatid cohesion and cohesins play a key role in promoting kinetochore orientation and proper chromosome segregation [7,134,135]. As described above, in *S. cerevisiae* it is proposed that early replication timing is also required for proper kinetochore orientation, although the role of cohesin has not been verified [58,89,90].

The details of cohesion establishment and subsequent removal are well reviewed elsewhere [7,133,134]. Briefly, during the S phase the cohesin complex is loaded and activated to cohere to newly duplicated sister chromatids together. Replication fork passage is accompanied by acetylation of the cohesion complex, which stabilizes its association. During prophase, arm cohesion is removed in most organisms; centromere cohesin undergoes proteolytic cleavage during anaphase to allow the sister chromatids to complete their separation.

Components of the replisome are linked to cohesion establishment in fission yeast, including the coupling proteins Swi1, Swi3, and Mcl1 [136,137], and there is evidence for an association with core components of the replication fork, such as MCMs in other systems [138,139,140,141,142]. Cohesion at the centromere additionally depends upon Swi6 [22,23] and is mediated in part by DDK, which promotes cohesin phosphorylation [24]. Intriguingly, this requirement for Swi6 in cohesion can be genetically separated from the role of Swi6 in heterochromatin formation [143]. This separation-of-function analysis indicates that chromosome segregation defects associated with a loss of Swi6 reflect a loss of centromere cohesion rather than defects in transcriptional silencing in this domain.

Replicating chromatids are also physically entangled by sister chromatid intertwinings (SCI) that occur as a consequence of replication progression (reviewed in [133,134,144]). This may reflect regions of unreplicated DNA or, more commonly, entangled sister chromatids or catenanes that require resolution by topoisomerase II. Importantly, one class of SCI is detected between sister centromeres and visualized as ultra-fine anaphase bridges (UFBs) [145]. These threads of ssDNA cannot be seen with typical DNA intercalating dyes or with histone labels but can be visualized by binding by ssDNA binding proteins, including RPA and the BLM helicase [47,48,59,146]. There is evidence that UFBs are linked to under-replicated DNA at fragile sites (e.g., [4,48]), but evidence also suggests that the centromere-associated UFBs are a normal feature of mitosis (reviewed in [145]). Increased UFBs in fission yeast are observed in mutants that suffer replication stress, although it is not clear whether these are centromere-associated [47,59].

Catenanes are preserved by the presence of cohesion because their resolution correlates to decreased cohesion, particularly on the arms [147,148,149,150]. Recent studies suggest that bidirectional topoisomerase activity continues during G2/M on cohered chromosomes, allowing both increased and decreased entanglements [151]. Driving the reaction to favor decatenation depends upon cohesin removal, as well as chromosome condensation at anaphase [148,151,152,153].

In addition to linking sister chromatids and contributing to centromere function, cohesin also plays key roles in organizing the genome for DNA replication, in responding to replication stress, and in facilitating DNA repair in multiple systems (e.g., [138,154,155,156,157,158,159]). The natural instability of the pericentromere repeats, described above, may also facilitate the recruitment of cohesin and be one means of linking replication stress to heterochromatin, as proposed in [105].

## 5. Conclusions

The pericentromeres in *S. pombe* contain long tracts of repeated sequences that are protected by classic heterochromatin, including histone H3K9 methylation and the binding of Swi6, a homologue of heterochromatin protein 1 (HP-1). This structure is similar to that observed in mammalian cells. The heterochromatin is cyclically disrupted during mitosis and re-formed during DNA replication. Evidence suggests that these repeated sequences are intrinsically unstable, as indicated by increased levels of histone H2A(X) phosphorylation [93]. A simple model suggests that the assembly of heterochromatin protects the repeats from rearrangement during the S phase. Swi6 is required for early replication timing in the pericentromere, at least in part by the recruitment of the DDK replication initiation kinase [19,80]. In addition to causing late centromere replication, *swi6∆* cells are particularly sensitive to loss of the fork protection complex, and double mutants are prone to rearrangements [14]. However, early replication is not sufficient to maintain genome stability in this domain; early replication also occurs in *clr4∆* mutants, yet these are also sensitive to loss of the FPC [14,19,80]. This suggests that some function associated with Swi6, and not limited to early replication, is important to maintain stability in the pericentromere.

Transcriptional repression in the pericentromere, associated with heterochromatin, may limit the potential for collisions between the replication and transcription apparatus ([82]; reviewed in [160,161]). This depends upon the RNAi mechanism, but *dcr1∆* mutants do not destabilize the pericentromere to the same extent as *swi6∆* [14], suggesting this is not the primary agent of instability. Another mechanism that may contribute is the recruitment of cohesin, which depends on Swi6 (and thus, Clr4) but is independent of Swi6’s silencing function [22,23,143]. Consistent with this, DDK and the replisome associated proteins of the FPC and Mcl1 are also associated with the proper activation of cohesin (e.g., [24,136,137]). However, rearrangements in the pericentromere domain do not occur in *mis4* mutants that have reduced cohesion [14,162], although that could be a limitation of the allele examined. Resolution of SCIs is a third candidate mechanism that may be disrupted in *swi6∆* cells and promote instability; more detailed cytological analysis and an investigation of topoisomerase II dynamics will be required to investigate this possibility. Finally, defects in replication fork pausing, which in some regions depend on the FPC [163,164], may exacerbate the instability of Swi6-deficieint pericentromere repeats. Heterochromatin spreading is partly limited by tRNA genes, which are known to contribute to fork pausing [101,102,103,104]. The intriguing overlap of the fork termination protein Sap1 and Cenp-B homologues in the limiting rearrangement of LTR repeats elsewhere in the genome (e.g., [113,114]) and suggests that one function for the Cenp-B homologues at the centromere may be limiting rearrangements.

Recent studies have investigated the replication of repetitive sequences associated with human centromeres [165,166]. For example, enrichment of the ORC complex binds to alpha-satellite in the absence of CENP-B, indicating that CENP-B may regulate the replication of centromeric regions [165]. Particularly intriguing is that Aze et al. [166] used artificial chromosomes in a *Xenopus* system to examine the replication of repetitive elements of centromeric DNA of human chromosome 17. These sequences showed enrichment of DNA repair factors, including the MSH2/MSH6 complex, MRN, and Mus81, as well as condensin. Significantly, they observed reduced binding of RPA and TopBP1, both in unperturbed cells and under conditions of replication stress, leading to reduced checkpoint activation. This reduced activation correlates with the formation of topoisomerase-dependent DNA loops, suggesting that more complex structures contribute to stability of the centromere domain.

These observations suggest that understanding how replication dynamics in the fission yeast pericentromere contribute to maintaining genome stability in a natural fragile site is likely to have relevance for centromere function in mammalian systems as well.

## References

[1] Verdaasdonk J.S., Bloom K. (2011). Centromeres: Unique chromatin structures that drive chromosome segregation. Nat. Rev. Mol. Cell Biol..

[2] Slee R.B., Steiner C.M., Herbert B.S., Vance G.H., Hickey R.J., Schwarz T., Christan S., Radovich M., Schneider B.P., Schindelhauer D. (2012). Cancer-associated alteration of pericentromeric heterochromatin may contribute to chromosome instability. Oncogene.

[3] Martinez A.C., van Wely K.H. (2011). Centromere fission, not telomere erosion, triggers chromosomal instability in human carcinomas. Carcinogenesis.

[4] Beeharry N., Rattner J.B., Caviston J.P., Yen T. (2013). Centromere fragmentation is a common mitotic defect of S and G 2 checkpoint override. Cell Cycle.

[5] Janssen A., van der Burg M., Szuhai K., Kops G.J., Medema R.H. (2011). Chromosome segregation errors as a cause of DNA damage and structural chromosome aberrations. Science.

[6] Morris C.A., Moazed D. (2007). Centromere assembly and propagation. Cell.

[7] Tanaka T.U., Clayton L., Natsume T. (2013). Three wise centromere functions: See no error, hear no break, speak no delay. EMBO Rep..

[8] Voineagu I., Surka C.F., Shishkin A.A., Krasilnikova M.M., Mirkin S.M. (2009). Replisome stalling and stabilization at CGG repeats, which are responsible for chromosomal fragility. Nat. Struct. Mol. Biol..

[9] Voineagu I., Narayanan V., Lobachev K.S., Mirkin S.M. (2008). Replication stalling at unstable inverted repeats: Interplay between DNA hairpins and fork stabilizing proteins. Proc. Natl. Acad. Sci. USA.

[10] Mizuno K., Lambert S., Baldacci G., Murray J.M., Carr A.M. (2009). Nearby inverted repeats fuse to generate acentric and dicentric palindromic chromosomes by a replication template exchange mechanism. Genes Dev..

[11] Sofueva S., Du L.L., Limbo O., Williams J.S., Russell P. (2010). Brct domain interactions with phospho-histone H2A target Crb2 to chromatin at double-strand breaks and maintain the DNA damage checkpoint. Mol. Cell. Biol..

[12] Arlt M.F., Durkin S.G., Ragland R.L., Glover T.W. (2006). Common fragile sites as targets for chromosome rearrangements. DNA Repair (Amst).

[13] Alper B.J., Lowe B.R., Partridge J.F. (2012). Centromeric heterochromatin assembly in fission yeast—Balancing transcription, RNA interference and chromatin modification. Chromosome Res..

[14] Li P.C., Petreaca R.C., Jensen A., Yuan J.P., Green M.D., Forsburg S.L. (2013). Replication fork stability is essential for the maintenance of centromere integrity in the absence of heterochromatin. Cell Rep..

[15] Dialynas G.K., Vitalini M.W., Wallrath L.L. (2008). Linking heterochromatin protein 1 (HP1) to cancer progression. Mutat. Res..

[16] Morgan M.A., Shilatifard A. (2015). Chromatin signatures of cancer. Genes Dev..

[17] Kloc A., Zaratiegui M., Nora E., Martienssen R. (2008). RNA interference guides histone modification during the S phase of chromosomal replication. Curr. Biol..

[18] Chen E.S., Zhang K., Nicolas E., Cam H.P., Zofall M., Grewal S.I. (2008). Cell cycle control of centromeric repeat transcription and heterochromatin assembly. Nature.

[19] Hayashi M.T., Takahashi T.S., Nakagawa T., Nakayama J., Masukata H. (2009). The heterochromatin protein Swi6/HP1 activates replication origins at the pericentromeric region and silent mating-type locus. Nat. Cell Biol..

[20] Li P.C., Chretien L., Cote J., Kelly T.J., Forsburg S.L.S. (2011). Pombe replication protein Cdc18 (Cdc6) interacts with Swi6 (HP1) heterochromatin protein: Region specific effects and replication timing in the centromere. Cell Cycle.

[21] Kim S.M., Dubey D.D., Huberman J.A. (2003). Early-replicating heterochromatin. Genes Dev..

[22] Bernard P., Maure J.F., Partridge J.F., Genier S., Javerzat J.P., Allshire R.C. (2001). Requirement of heterochromatin for cohesion at centromeres. Science.

[23] Nonaka N., Kitajima T., Shihori Y., Xiao G., Yamamoto M., Grewal S.I., Watanabe Y. (2002). Recruitment of cohesin to heterochromatic regions by Swi6/HP1 in fission yeast. Nat. Cell Biol..

[24] Bailis J.M., Bernard P., Antonelli R., Allshire R., Forsburg S.L. (2003). Hsk1/Dfp1 is required for heterochromatin-mediated cohesion at centromeres. Nat. Cell Biol..

[25] Zhang D., O’Donnell M. (2016). The eukaryotic replication machine. Enzymes.

[26] Hills S.A., Diffley J.F. (2014). DNA replication and oncogene-induced replicative stress. Curr. Biol..

[27] Tanaka S., Araki H. (2010). Regulation of the initiation step of DNA replication by cyclin-dependent kinases. Chromosoma.

[28] Matsumoto S., Masai H. (2013). Regulation of chromosome dynamics by hsk1/cdc7 kinase. Biochem. Soc. Trans..

[29] Labib K., Gambus A. (2007). A key role for the GINS complex at DNA replication forks. Trends Cell Biol..

[30] Sun J., Shi Y., Georgescu R.E., Yuan Z., Chait B.T., Li H., O’Donnell M.E. (2015). The architecture of a eukaryotic replisome. Nat. Struct. Mol. Biol..

[31] Langston L.D., Zhang D., Yurieva O., Georgescu R.E., Finkelstein J., Yao N.Y., Indiani C., O’Donnell M.E. (2014). CMG helicase and DNA polymerase epsilon form a functional 15-subunit holoenzyme for eukaryotic leading-strand DNA replication. Proc. Natl. Acad. Sci. USA.

[32] Sabatinos S.A., Forsburg S.L. (2015). Managing single-stranded DNA during replication stress in fission yeast. Biomolecules.

[33] Alcasabas A.A., Osborn A.J., Bachant J., Hu F., Werler P.J., Bousset K., Furuya K., Diffley J.F., Carr A.M., Elledge S.J. (2001). MRC1 transduces signals of DNA replication stress to activate rad53. Nat. Cell Biol..

[34] Tanaka K., Russell P. (2001). MRC1 channels the DNA replication arrest signal to checkpoint kinase Cds1. Nat. Cell Biol..

[35] Leman A.R., Noguchi E. (2012). Local and global functions of Timeless and Tipin in replication fork protection. Cell Cycle.

[36] Simon A.C., Zhou J.C., Perera R.L., van Deursen F., Evrin C., Ivanova M.E., Kilkenny M.L., Renault L., Kjaer S., Matak-Vinkovic D. (2014). A ctf4 trimer couples the CMG helicase to DNA polymerase alpha in the eukaryotic replisome. Nature.

[37] Villa F., Simon A.C., Ortiz Bazan M.A., Kilkenny M.L., Wirthensohn D., Wightman M., Matak-Vinkovic D., Pellegrini L., Labib K. (2016). Ctf4 is a hub in the eukaryotic replisome that links multiple CIP-Box proteins to the cmg helicase. Mol. Cell.

[38] Zeman M.K., Cimprich K.A. (2014). Causes and consequences of replication stress. Nat. Cell Biol..

[39] Debatisse M., le Tallec B., Letessier A., Dutrillaux B., Brison O. (2012). Common fragile sites: Mechanisms of instability revisited. Trends Genet..

[40] Carr A.M., Lambert S. (2013). Replication stress-induced genome instability: The dark side of replication maintenance by homologous recombination. J. Mol. Biol..

[41] Macheret M., Halazonetis T.D. (2015). DNA replication stress as a hallmark of cancer. Annu. Rev. Pathol..

[42] Marechal A., Zou L. (2015). Rpa-coated single-stranded DNA as a platform for post-translational modifications in the DNA damage response. Cell Res..

[43] Byun T.S., Pacek M., Yee M.C., Walter J.C., Cimprich K.A. (2005). Functional uncoupling of MCM helicase and DNA polymerase activities activates the ATR-dependent checkpoint. Genes Dev..

[44] Lopes M., Foiani M., Sogo J.M. (2006). Multiple mechanisms control chromosome integrity after replication fork uncoupling and restart at irreparable UV lesions. Mol. Cell.

[45] Hu J., Sun L., Shen F., Chen Y., Hua Y., Liu Y., Zhang M., Hu Y., Wang Q., Xu W. (2012). The intra-S phase checkpoint targets DNA2 to prevent stalled replication forks from reversing. Cell.

[46] Ira G., Pellicioli A., Balijja A., Wang X., Fiorani S., Carotenuto W., Liberi G., Bressan D., Wan L., Hollingsworth N.M. (2004). DNA end resection, homologous recombination and DNA damage checkpoint activation require cdk1. Nature.

[47] Sofueva S., Osman F., Lorenz A., Steinacher R., Castagnetti S., Ledesma J., Whitby M.C. (2011). Ultrafine anaphase bridges, broken DNA and illegitimate recombination induced by a replication fork barrier. Nucleic Acids Res..

[48] Chan K.L., Palmai-Pallag T., Ying S., Hickson I.D. (2009). Replication stress induces sister-chromatid bridging at fragile site loci in mitosis. Nat. Cell Biol.

[49] Roberts S.A., Sterling J., Thompson C., Harris S., Mav D., Shah R., Klimczak L.J., Kryukov G.V., Malc E., Mieczkowski P.A. (2012). Clustered mutations in yeast and in human cancers can arise from damaged long single-strand DNA regions. Mol. Cell.

[50] Fanning E., Klimovich V., Nager A.R. (2006). A dynamic model for replication protein a (RPA) function in DNA processing pathways. Nucleic Acids Res..

[51] Toledo L.I., Altmeyer M., Rask M.B., Lukas C., Larsen D.H., Povlsen L.K., Bekker-Jensen S., Mailand N., Bartek J., Lukas J. (2013). ATR prohibits replication catastrophe by preventing global exhaustion of RPA. Cell.

[52] Branzei D., Foiani M. (2009). The checkpoint response to replication stress. DNA Repair (Amst).

[53] Branzei D., Foiani M. (2010). Maintaining genome stability at the replication fork. Nat. Rev. Mol. Cell Biol..

[54] Petermann E., Helleday T. (2010). Pathways of mammalian replication fork restart. Nat. Rev. Mol. Cell Biol..

[55] McIntosh D., Blow J.J. (2012). Dormant origins, the licensing checkpoint, and the response to replicative stresses. Cold Spring Harb. Perspect. Biol..

[56] Mizuno K., Miyabe I., Schalbetter S.A., Carr A.M., Murray J.M. (2013). Recombination-restarted replication makes inverted chromosome fusions at inverted repeats. Nature.

[57] Minocherhomji S., Ying S., Bjerregaard V.A., Bursomanno S., Aleliunaite A., Wu W., Mankouri H.W., Shen H., Liu Y., Hickson I.D. (2015). Replication stress activates DNA repair synthesis in mitosis. Nature.

[58] Feng W., Bachant J., Collingwood D., Raghuraman M.K., Brewer B.J. (2009). Centromere replication timing determines different forms of genomic instability in saccharomyces cerevisiae checkpoint mutants during replication stress. Genetics.

[59] Sabatinos S.A., Ranatunga N.S., Yuan J.P., Green M.D., Forsburg S.L. (2015). Replication stress in early S phase generates apparent micronuclei and chromosome rearrangement in fission yeast. Mol. Biol. Cell.

[60] Allshire R.C., Karpen G.H. (2008). Epigenetic regulation of centromeric chromatin: Old dogs, new tricks?. Nat. Rev. Genet..

[61] Wood V., Gwilliam R., Rajandream M.A., Lyne M., Lyne R., Stewart A., Sgouros J., Peat N., Hayles J., Baker S. (2002). The genome sequence of schizosaccharomyces pombe. Nature.

[62] Nakayama J., Rice J.C., Strahl B.D., Allis C.D., Grewal S.I. (2001). Role of histone H3 lysine 9 methylation in epigenetic control of heterochromatin assembly. Science.

[63] Rea S., Eisenhaber F., O’Carroll D., Strahl B.D., Sun Z.W., Schmid M., Opravil S., Mechtler K., Ponting C.P., Allis C.D. (2000). Regulation of chromatin structure by site-specific histone H3 methyltransferases. Nature.

[64] Noma K., Sugiyama T., Cam H., Verdel A., Zofall M., Jia S., Moazed D., Grewal S.I. (2004). Rits acts in Cis to promote RNA interference-mediated transcriptional and post-transcriptional silencing. Nat. Genet..

[65] Verdel A., Jia S., Gerber S., Sugiyama T., Gygi S., Grewal S.I., Moazed D. (2004). RNAi-mediated targeting of heterochromatin by the RITS complex. Science.

[66] Schalch T., Job G., Shanker S., Partridge J.F., Joshua-Tor L. (2011). The Chp1-Tas3 core is a multifunctional platform critical for gene silencing by RITS. Nat. Struct. Mol. Biol..

[67] Schalch T., Job G., Noffsinger V.J., Shanker S., Kuscu C., Joshua-Tor L., Partridge J.F. (2009). High-affinity binding of Chp1 chromodomain to K9 methylated histone H3 is required to establish centromeric heterochromatin. Mol. Cell.

[68] Hayashi A., Ishida M., Kawaguchi R., Urano T., Murakami Y., Nakayama J. (2012). Heterochromatin protein 1 homologue swi6 acts in concert with Ers1 to regulate rnai-directed heterochromatin assembly. Proc. Natl. Acad. Sci. USA.

[69] Sadaie M., Iida T., Urano T., Nakayama J. (2004). A chromodomain protein, Chp1, is required for the establishment of heterochromatin in fission yeast. EMBO J..

[70] Debeauchamp J.L., Moses A., Noffsinger V.J., Ulrich D.L., Job G., Kosinski A.M., Partridge J.F. (2008). Chp1-tas3 interaction is required to recruit RITS to fission yeast centromeres and for maintenance of centromeric heterochromatin. Mol. Cell. Biol..

[71] Zhang K., Mosch K., Fischle W., Grewal S.I. (2008). Roles of the clr4 methyltransferase complex in nucleation, spreading and maintenance of heterochromatin. Nat. Struct. Mol. Biol..

[72] Hall I.M., Shankaranarayana G.D., Noma K., Ayoub N., Cohen A., Grewal S.I. (2002). Establishment and maintenance of a heterochromatin domain. Science.

[73] Yamamoto K., Sonoda M. (2003). Self-interaction of heterochromatin protein 1 is required for direct binding to histone methyltransferase, SUV39H1. Biochem. Biophys. Res. Commun..

[74] Li F., Martienssen R., Cande W.Z. (2011). Coordination of DNA replication and histone modification by the rik1-dos2 complex. Nature.

[75] Lejeune E., Bayne E.H., Allshire R.C. (2010). On the connection between RNAi and heterochromatin at centromeres. Cold Spring Harb. Symp. Quant. Biol..

[76] Doe C.L., Wang G., Chow C.-M., Fricker M.D., Singh P.B., Mellor E.J. (1998). The fission yeast chromo domain encoding gene *chp1^+^* is required for chromosome segregation and shows a genetic interaction with alpha-tubulin. Nucleic Acids Res..

[77] Ekwall K., Javerzat J.P., Lorentz A., Schmidt H., Cranston G., Allshire R. (1995). The chromodomain protein Swi6: A key component at fission yeast centromeres. Science.

[78] Ekwall K., Nimmo E.R., Javerzat J.P., Borgstrom B., Egel R., Cranston G., Allshire R. (1996). Mutations in the fission yeast silencing factors Clr4+ and Rik1+ disrupt the localisation of the chromo domain protein Swi6p and impair centromere function. J. Cell Sci..

[79] Thon G., Verhein-Hansen J. (2000). Four chromo-domain proteins of *schizosaccharmyces pombe* differentially repress transcription at various chromosomal locations. Genetics.

[80] Li P.C., Green M.D., Forsburg S.L. (2013). Mutations disrupting histone methylation have different effects on replication timing in S. Pombe centromere. PLoS ONE.

[81] Wohlgemuth J.G., Bulboaca G.H., Moghadam M., Caddle M.S., Calos M.P. (1994). Physical mapping of origins of replication in the fission yeast *schizosaccharomyces pombe*. Mol. Biol. Cell.

[82] Zaratiegui M., Castel S.E., Irvine D.V., Kloc A., Ren J., Li F., de Castro E., Marin L., Chang A.Y., Goto D. (2011). RNAi promotes heterochromatic silencing through replication-coupled release of RNA pol ii. Nature.

[83] Nakayama J., Allshire R.C., Klar A.J., Grewal S.I. (2001). A role for DNA polymerase alpha in epigenetic control of transcriptional silencing in fission yeast. EMBO J..

[84] Dohke K., Miyazaki S., Tanaka K., Urano T., Grewal S.I., Murakami Y. (2008). Fission yeast chromatin assembly factor 1 assists in the replication-coupled maintenance of heterochromatin. Genes Cells.

[85] Motamedi M.R., Hong E.J., Li X., Gerber S., Denison C., Gygi S., Moazed D. (2008). Hp1 proteins form distinct complexes and mediate heterochromatic gene silencing by nonoverlapping mechanisms. Mol. Cell.

[86] Sugiyama T., Cam H., Verdel A., Moazed D., Grewal S.I. (2005). RNA-dependent RNA polymerase is an essential component of a self-enforcing loop coupling heterochromatin assembly to sirna production. Proc. Natl. Acad. Sci. USA.

[87] Forsburg S.L. (2013). The CINS of the centromere. Biochem. Soc. Trans..

[88] Natsume T., Tsutsui Y., Sutani T., Dunleavy E.M., Pidoux A.L., Iwasaki H., Shirahige K., Allshire R.C., Yamao F. (2008). A DNA polymerase alpha accessory protein, Mcl1, is required for propagation of centromere structures in fission yeast. PLoS ONE.

[89] Bachant J., Jessen S.R., Kavanaugh S.E., Fielding C.S. (2005). The yeast s phase checkpoint enables replicating chromosomes to bi-orient and restrain spindle extension during s phase distress. J. Cell Biol..

[90] Natsume T., Muller C.A., Katou Y., Retkute R., Gierlinski M., Araki H., Blow J.J., Shirahige K., Nieduszynski C.A., Tanaka T.U. (2013). Kinetochores coordinate pericentromeric cohesion and early DNA replication by Cdc7-Dbf4 kinase recruitment. Mol. Cell.

[91] Deng W., Tsao S.W., Guan X.Y., Cheung A.L. (2012). Pericentromeric regions are refractory to prompt repair after replication stress-induced breakage in HPV16 E6E7-expressing epithelial cells. PLoS ONE.

[92] Simi S., Simili M., Bonatti S., Campagna M., Abbondandolo A. (1998). Fragile sites at the centromere of chinese hamster chromosomes: A possible mechanism of chromosome loss. Mutat. Res..

[93] Rozenzhak S., Mejia-Ramirez E., Williams J.S., Schaffer L., Hammond J.A., Head S.R., Russell P. (2010). Rad3 decorates critical chromosomal domains with gammah2a to protect genome integrity during S-phase in fission yeast. PLoS Genet..

[94] Burma S., Chen B.P., Murphy M., Kurimasa A., Chen D.J. (2001). Atm phosphorylates histone H2AX in response to DNA double-strand breaks. J. Biol. Chem..

[95] Nakamura T.M., Du L.L., Redon C., Russell P. (2004). Histone H2A phosphorylation controls Crb2 recruitment at DNA breaks, maintains checkpoint arrest, and influences DNA repair in fission yeast. Mol. Cell. Biol..

[96] Irmisch A., Ampatzidou E., Mizuno K., O’Connell M.J., Murray J.M. (2009). Smc5/6 maintains stalled replication forks in a recombination-competent conformation. EMBO J..

[97] Pebernard S., Schaffer L., Campbell D., Head S.R., Boddy M.N. (2008). Localization of Smc5/6 to centromeres and telomeres requires heterochromatin and sumo, respectively. Embo J..

[98] Tapia-Alveal C., O’Connell M.J. (2011). Nse1-dependent recruitment of Smc5/6 to lesion-containing loci contributes to the repair defects of mutant complexes. Mol. Biol. Cell.

[99] Ampatzidou E., Irmisch A., O’Connell M.J., Murray J.M. (2006). Smc5/6 is required for repair at collapsed replication forks. Mol. Cell. Biol..

[100] Lee S.Y., Rozenzhak S., Russell P. (2013). Gammah2a-binding protein Brc1 affects centromere function in fission yeast. Mol. Cell. Biol..

[101] Kuhn R.M., Clarke L., Carbon J. (1991). Clustered tRNA genes in schizosaccharomyces pombe centromeric DNA sequence repeats. Proc. Natl. Acad. Sci. USA.

[102] Iwasaki O., Tanaka A., Tanizawa H., Grewal S.I., Noma K. (2010). Centromeric localization of dispersed pol III genes in fission yeast. Mol. Biol. Cell.

[103] Scott K.C., Merrett S.L., Willard H.F. (2006). A heterochromatin barrier partitions the fission yeast centromere into discrete chromatin domains. Curr. Biol..

[104] Deshpande A.M., Newlon C.S. (1996). Dna replication fork pause sites dependent on transcription. Science.

[105] Nikolov I., Taddei A. (2016). Linking replication stress with heterochromatin formation. Chromosoma.

[106] Greenfeder S.A., Newlon C.S. (1992). Replication forks pause at yeast centromeres. Mol. Cell. Biol..

[107] Baum M., Clarke L. (2000). Fission yeast homologs of human Cenp-B have redundant functions affecting cell growth and chromosome segregation. Mol. Cell. Biol..

[108] Nakagawa H., Lee J.K., Hurwitz J., Allshire R.C., Nakayama J., Grewal S.I., Tanaka K., Murakami Y. (2002). Fission yeast Cenp-B homologs nucleate centromeric heterochromatin by promoting heterochromatin-specific histone tail modifications. Genes Dev..

[109] Casola C., Hucks D., Feschotte C. (2008). Convergent domestication of pogo-like transposases into centromere-binding proteins in fission yeast and mammals. Mol. Biol. Evol..

[110] Murakami Y., Huberman J.A., Hurwitz J. (1996). Identification, purification, and molecular cloning of autonomously replicating sequence-binding protein 1 from fission yeast *schizosaccharomyces pombe*. Proc. Natl. Acad. Sci. USA.

[111] Irelan J.T., Gutkin G.I., Clarke L. (2001). Functional redundancies, distinct localizations and interactions among three fission yeast homologs of centromere protein-B. Genetics.

[112] Lee J.K., Huberman J.A., Hurwitz J. (1997). Purification and characterization of a Cenp-B homologue protein that binds to the centromeric k-type repeat DNA of *schizosaccharomyces pombe*. Proc. Natl. Acad. Sci. USA.

[113] Zaratiegui M., Vaughn M.W., Irvine D.V., Goto D., Watt S., Bahler J., Arcangioli B., Martienssen R.A. (2011). Cenp-B preserves genome integrity at replication forks paused by retrotransposon ltr. Nature.

[114] Johansen P., Cam H.P. (2015). Suppression of meiotic recombination by Cenp-B homologs in schizosaccharomyces pombe. Genetics.

[115] Krings G., Bastia D. (2005). Sap1p binds to TER1 at the ribosomal DNA of schizosaccharomyces pombe and causes polar replication fork arrest. J. Biol. Chem..

[116] Mejia-Ramirez E., Sanchez-Gorostiaga A., Krimer D.B., Schvartzman J.B., Hernandez P. (2005). The mating type switch-activating protein Sap1 is required for replication fork arrest at the rRNA genes of fission yeast. Mol. Cell. Biol..

[117] Arcangioli B., Copeland T.D., Klar A.J.S. (1994). Sap1, a protein that binds to sequences required for mating-type switching, is essential for viability in *schizosaccharomyces pombe*. Mol. Cell. Biol..

[118] De Lahondes R., Ribes V., Arcangioli B. (2003). Fission yeast Sap1 protein is essential for chromosome stability. Eukaryot Cell..

[119] Noguchi C., Noguchi E. (2007). Sap1 promotes the association of the replication fork protection complex with chromatin and is involved in the replication checkpoint in schizosaccharomyces pombe. Genetics.

[120] Gadaleta M.C., Das M.M., Tanizawa H., Chang Y.T., Noma K., Nakamura T.M., Noguchi E. (2016). Swi1timeless prevents repeat instability at fission yeast telomeres. PLoS Genet..

[121] Nakaseko Y., Kinoshita N., Yanagida M. (1987). A novel sequence common to the centromere regions of schizosaccharomyces pombe chromosomes. Nucleic Acids Res..

[122] Ellermeier C., Higuchi E.C., Phadnis N., Holm L., Geelhood J.L., Thon G., Smith G.R. (2010). RNAi and heterochromatin repress centromeric meiotic recombination. Proc. Natl. Acad. Sci. USA.

[123] Vincenten N., Kuhl L.M., Lam I., Oke A., Kerr A.R., Hochwagen A., Fung J., Keeney S., Vader G., Marston A.L. (2015). The kinetochore prevents centromere-proximal crossover recombination during meiosis. Elife.

[124] Takahashi K., Murakami S., Chikashige Y., Funabiki H., Niwa O., Yanagida M. (1992). A low copy number central sequence with strict symmetry and unusual chromatin structure in fission yeast centromere. Mol. Biol. Cell.

[125] Pidoux A.L., Allshire R.C. (2005). The role of heterochromatin in centromere function. Philos. Trans. R. Soc. Lond. B Biol. Sci..

[126] McFarlane R.J., Humphrey T.C. (2010). A role for recombination in centromere function. Trends Genet..

[127] Tinline-Purvis H., Savory A.P., Cullen J.K., Dave A., Moss J., Bridge W.L., Marguerat S., Bahler J., Ragoussis J., Mott R. (2009). Failed gene conversion leads to extensive end processing and chromosomal rearrangements in fission yeast. EMBO J..

[128] Nakamura K., Okamoto A., Katou Y., Yadani C., Shitanda T., Kaweeteerawat C., Takahashi T.S., Itoh T., Shirahige K., Masukata H. (2008). Rad51 suppresses gross chromosomal rearrangement at centromere in schizosaccharomyces pombe. EMBO J..

[129] Onaka A.T., Toyofuku N., Inoue T., Okita A.K., Sagawa M., Su J., Shitanda T., Matsuyama R., Zafar F., Takahashi T.S. (2016). Rad51 and Rad54 promote noncrossover recombination between centromere repeats on the same chromatid to prevent isochromosome formation. Nucleic Acids Res..

[130] Naim V., Wilhelm T., Debatisse M., Rosselli F. (2013). Ercc1 and Mus81-Eme1 promote sister chromatid separation by processing late replication intermediates at common fragile sites during mitosis. Nat. Cell Biol..

[131] Ying S., Minocherhomji S., Chan K.L., Palmai-Pallag T., Chu W.K., Wass T., Mankouri H.W., Liu Y., Hickson I.D. (2013). Mus81 promotes common fragile site expression. Nat. Cell Biol..

[132] Roguev A., Bandyopadhyay S., Zofall M., Zhang K., Fischer T., Collins S.R., Qu H., Shales M., Park H.O., Hayles J. (2008). Conservation and rewiring of functional modules revealed by an epistasis map in fission yeast. Science.

[133] Hirano T. (2015). Chromosome dynamics during mitosis. Cold Spring Harb. Perspect. Biol..

[134] Haarhuis J.H., Elbatsh A.M., Rowland B.D. (2014). Cohesin and its regulation: On the logic of X-shaped chromosomes. Dev. Cell.

[135] Nasmyth K., Haering C.H. (2009). Cohesin: Its roles and mechanisms. Annu. Rev. Genet..

[136] Ansbach A.B., Noguchi C., Klansek I.W., Heidlebaugh M., Nakamura T.M., Noguchi E. (2008). Rfcctf18 and the swi1-swi3 complex function in separate and redundant pathways required for the stabilization of replication forks to facilitate sister chromatid cohesion in schizosaccharomyces pombe. Mol. Biol. Cell.

[137] Williams D.R., McIntosh J.R. (2002). Mcl1+, the schizosaccharomyces pombe homologue of Ctf4, is important for chromosome replication, cohesion, and segregation. Eukaryot. Cell.

[138] Guillou E., Ibarra A., Coulon V., Casado-Vela J., Rico D., Casal I., Schwob E., Losada A., Mendez J. (2010). Cohesin organizes chromatin loops at DNA replication factories. Genes Dev..

[139] Panigrahi A.K., Zhang N., Otta S.K., Pati D. (2012). A cohesin-rad21 interactome. Biochem. J..

[140] Unal E., Heidinger-Pauli J.M., Koshland D. (2007). DNA double-strand breaks trigger genome-wide sister-chromatid cohesion through eco1 (ctf7). Science.

[141] Unal E., Arbel-Eden A., Sattler U., Shroff R., Lichten M., Haber J.E., Koshland D. (2004). DNA damage response pathway uses histone modification to assemble a double-strand break-specific cohesin domain. Mol. Cell.

[142] Ryu M.J., Kim B.J., Lee J.W., Lee M.W., Choi H.K., Kim S.T. (2006). Direct interaction between cohesin complex and DNA replication machinery. Biochem. Biophys. Res. Commun..

[143] Yamagishi Y., Sakuno T., Shimura M., Watanabe Y. (2008). Heterochromatin links to centromeric protection by recruiting shugoshin. Nature.

[144] Baxter J. (2015). “Breaking up is hard to do”: The formation and resolution of sister chromatid intertwines. J. Mol. Biol..

[145] Liu Y., Nielsen C.F., Yao Q., Hickson I.D. (2014). The origins and processing of ultra fine anaphase DNA bridges. Curr. Opin. Genet. Dev..

[146] Burrell R.A., McClelland S.E., Endesfelder D., Groth P., Weller M.C., Shaikh N., Domingo E., Kanu N., Dewhurst S.M., Gronroos E. (2013). Replication stress links structural and numerical cancer chromosomal instability. Nature.

[147] Farcas A.M., Uluocak P., Helmhart W., Nasmyth K. (2011). Cohesin’s concatenation of sister dnas maintains their intertwining. Mol. Cell.

[148] Wang L.H., Mayer B., Stemmann O., Nigg E.A. (2010). Centromere DNA decatenation depends on cohesin removal and is required for mammalian cell division. J. Cell Sci..

[149] Oliveira R.A., Hamilton R.S., Pauli A., Davis I., Nasmyth K. (2010). Cohesin cleavage and cdk inhibition trigger formation of daughter nuclei. Nat. Cell Biol..

[150] Haarhuis J.H., Elbatsh A.M., van den Broek B., Camps D., Erkan H., Jalink K., Medema R.H., Rowland B.D. (2013). Wapl-mediated removal of cohesin protects against segregation errors and aneuploidy. Curr. Biol..

[151] Sen N., Leonard J., Torres R., Garcia-Luis J., Palou-Marin G., Aragon L. (2016). Physical proximity of sister chromatids promotes top2-dependent intertwining. Mol. Cell.

[152] Charbin A., Bouchoux C., Uhlmann F. (2014). Condensin aids sister chromatid decatenation by topoisomerase ii. Nucleic Acids Res..

[153] Coelho P.A., Queiroz-Machado J., Sunkel C.E. (2003). Condensin-dependent localisation of topoisomerase ii to an axial chromosomal structure is required for sister chromatid resolution during mitosis. J. Cell Sci..

[154] Tittel-Elmer M., Lengronne A., Davidson M.B., Bacal J., Francois P., Hohl M., Petrini J.H., Pasero P., Cobb J.A. (2012). Cohesin association to replication sites depends on rad50 and promotes fork restart. Mol. Cell.

[155] Sjögren C., Nasmyth K. (2001). Sister chromatid cohesion is required for postreplicative double-strand break repair in *saccharomyces cerevisiae*. Curr. Biol..

[156] Tatebayashi K., Kato J., Ikeda H. (1998). Isolation of a schizosaccharomyces pombe rad21ts mutant that is aberrant in chromosome segregation, microtubule function, DNA repair and sensitive to hydroxyurea: Possible involvement of rad21 in ubiquitin-mediated proteolysis. Genetics.

[157] Birkenbihl R.P., Subramani S. (1992). Cloning and characterization of rad21 an essential gene of schizosaccharomyces pombe involved in DNA double-strand-break repair. Nucliec Acids Res..

[158] Cortes-Ledesma F., Aguilera A. (2006). Double-strand breaks arising by replication through a nick are repaired by cohesin-dependent sister-chromatid exchange. EMBO Rep..

[159] Gelot C., Guirouilh-Barbat J., Lopez B.S. (2016). The cohesin complex prevents the end-joining of distant DNA double-strand ends in s phase: Consequences on genome stability maintenance. Nucleus.

[160] Hamperl S., Cimprich K.A. (2016). Conflict resolution in the genome: How transcription and replication make it work. Cell.

[161] Garcia-Muse T., Aguilera A. (2016). Transcription-replication conflicts: How they occur and how they are resolved. Nat. Rev. Mol. Cell Biol..

[162] Furuya K., Takahashi K., Yanagida M. (1998). Faithful anaphase is endured by Mis4, a sister chromatid cohesion molecule required in sphase and not destroyed N G_1_ phase. Genes Dev..

[163] Dalgaard J.Z., Klar A.J.S. (2000). *Swi1* and *swi3* perform imprinting, pausing and termination of DNA replication in *S. Pombe*. Cell.

[164] Krings G., Bastia D. (2004). Swi1- and Swi3-dependent and independent replication fork arrest at the ribosomal DNA of schizosaccharomyces pombe. Proc. Natl. Acad. Sci. USA.

[165] Erliandri I., Fu H., Nakano M., Kim J.H., Miga K.H., Liskovykh M., Earnshaw W.C., Masumoto H., Kouprina N., Aladjem M.I. (2014). Replication of alpha-satellite DNA arrays in endogenous human centromeric regions and in human artificial chromosome. Nucleic Acids Res..

[166] Aze A., Sannino V., Soffientini P., Bachi A., Costanzo V. (2016). Centromeric DNA replication reconstitution reveals DNA loops and ATR checkpoint suppression. Nat. Cell Biol..

